# Users’ Experiences of a Mobile Health Self-Management Approach for the Treatment of Cystic Fibrosis: Mixed Methods Study

**DOI:** 10.2196/15896

**Published:** 2020-07-08

**Authors:** Jacqueline Floch, Thomas Vilarinho, Annabel Zettl, Gema Ibanez-Sanchez, Joaquim Calvo-Lerma, Erlend Stav, Peter Halland Haro, Asbjørn Lein Aalberg, Alvaro Fides-Valero, José Luis Bayo Montón

**Affiliations:** 1 SINTEF Trondheim Norway; 2 YOUSE GmbH München Germany; 3 Universitat Politècnica de València València Spain; 4 Instituto de Investigación Sanitaria La Fe València Spain

**Keywords:** mobile health, mHealth, self-management, user experience, user acceptance, mixed methods study, cystic fibrosis, pediatrics

## Abstract

**Background:**

Despite a large number of clinical trials aiming at evaluating the digital self-management of chronic diseases, there is little discussion about users’ experiences with digital approaches. However, a good user experience is a critical factor for technology adoption. Understanding users’ experiences can inform the design of approaches toward increased motivation for digital self-management.

**Objective:**

This study aimed to evaluate the self-management of cystic fibrosis (CF) with a focus on gastrointestinal concerns and the care of young patients. Following a user-centered design approach, we developed a self-management app for patients and parents and a web tool for health care professionals (HCPs). To evaluate the proposed solutions, a 6-month clinical trial was conducted in 6 European CF competence centers. This paper analyzes the user acceptance of the technology and the benefits and disadvantages perceived by the trial participants.

**Methods:**

A mixed methods approach was applied. Data were collected through 41 semistructured qualitative interviews of patients, parents, and HCPs involved in the clinical trial. In addition, data were collected through questionnaires embedded in the self-management app.

**Results:**

Support for enzyme dose calculation and nutrition management was found to be particularly useful. Patients and parents rapidly strengthened their knowledge about the treatment and increased their self-efficacy. Reported benefits include reduced occurrence of symptoms and enhanced quality of life. Patients and parents had different skills, requiring follow-up by HCPs in an introductory phase. HCPs valued obtaining precise information about the patients, allowing for more personalized advice. However, the tight follow-up of several patients led to an increased workload. Over time, as patient self-efficacy increased, patient motivation for using the app decreased and the quality of the reported data was reduced.

**Conclusions:**

Self-management enfolds a collaboration between patients and HCPs. To be successful, a self-management approach should be accepted by both parties. Through understanding behaviors and experiences, this study defines recommendations for a complex case—the demanding treatment of CF. We identify target patient groups and situations for which the app is most beneficial and suggest focusing on these rather than motivating for regular app usage over a long time. We also advise the personalized supervision of patients during the introduction of the approach. Finally, we propose to develop guidance for HCPs to facilitate changes in practice. As personalization and technology literacy are factors found to influence the acceptance of digital self-management of other chronic diseases, it is relevant to consider the proposed recommendations beyond the case of CF.

## Introduction

### Background

Chronic disease self-management enfolds the idea that patients in collaboration with health care professionals (HCPs) gain knowledge about the disease and carry out part of the treatment themselves [1]. Several reviews have observed a positive effect of digital self-management approaches [2-4]. Despite several clinical trials evaluating the digital self-management of chronic diseases, there is little discussion about users’ experiences with digital approaches. A good user experience, for example, making the technology useful, easy to use, and efficient, is a critical factor for technology adoption [5]. Understanding this experience can inform the design of approaches toward motivation for digital self-management. Both the viewpoints of patients and HCPs are essential for a successful collaboration.

The scope of our research is the self-management of cystic fibrosis (CF), a congenital, chronic disorder affecting the digestive and respiratory systems, resulting in malnutrition and respiratory infections [6]. Daily treatment is demanding, including physiotherapy, physical exercise, an adapted rich diet, and dosage of pancreatic enzyme supplements. Many tend not to adhere to the whole therapy [7]. Therefore, digital support that facilitates understanding of the treatment and motivates adherence is relevant but challenging.

All countries involved in this study have specialized CF centers [8]. The patients meet for consultation at least every 3 months. Between consultations, patients can contact their centers if needed. A thorough control is performed yearly, requiring a written collection of food records. Although the CF centers function well and patients express satisfaction in the services, our earlier research indicates the readiness for digital self-management in the CF care [9].

### MyCyFAPP Vision

Our research is part of the European Union–funded research project MyCyFAPP [10], aiming to increase patients’ knowledge regarding their treatment, facilitating adherence, and supporting teenagers’ implications. Most CF patients have to follow a pancreatic enzyme replacement therapy, where enzymes are taken with each meal to help digest food. Wrong doses can cause malnutrition and gastrointestinal (GI) problems [11]. Before MyCyFAPP, there were no knowledge or tools to adjust the dose of enzymes. Rather, patients were recommended a fixed dose for each meal. A key novel component developed in MyCyFAPP is an algorithm for enzyme dose calculation [12].

### Digital Self-Management in MyCyFAPP

The digital support developed in MyCyFAPP includes a self-management app targeting parents of young children with CF and teenagers with CF and a professional web tool (PWT) targeting HCPs (Multimedia Appendix 1).

The app is available in the languages of the participating countries (ie, Dutch, Spanish, Italian, and Portuguese) and English. Its main features are as follows:

1. Calculation of a personalized enzyme dose depending on meal composition
2. Follow-up of food intake
3. Food recording as a basis for both enzyme dose calculation and nutrition management
4. Access to recommended country-specific dishes to correct specific nutritional imbalances [13]
5. Health diary for recording mood and GI symptoms
6. Educational handbook about the disease and the treatment with focus on nutrition.

The PWT provides an overview of the patients’ progress, mainly based on the recorded data using the self-management app. In addition, information is gathered during consultations, for example, weight and height. The monitored parameters are those included in the CF nutritional guidelines: nutrient intake, enzyme dose, and nutritional status [14]. HCPs can register health information, set nutritional goals, and send messages to patients.

### Research on User Experience in Mobile Health

A recent review of qualitative studies about patients’ perceptions and experiences of mobile health (mHealth) apps identified 38 scientific articles (2013-2018) related to app evaluation [15]. Most apps deal with chronic diseases and provide either access to information, communication with HCPs, peer support, or self-monitoring. No study has addressed the experiences of CF self-management. Unfortunately, the review did not assess whether evaluations were performed in controlled settings, whether HCPs were users of the technology, or the experiences of HCPs. Overall, the review finds that mHealth has great potential to engage and empower patients. Personalization, technology literacy, intrusiveness, information validity, security, and privacy appear to influence the acceptance of solutions, overlapping with identified issues in our initial research [9]. The design of MyCyFAPP digital solutions considers all these issues, as further discussed.

A motivation for that review was that mHealth apps seem to be underused after download, and app adherence is a major concern in consumer apps for health monitoring [16]. The review suggests a constant stimulation of patients to accommodate changing patients’ requirements. We argue that the purpose of mHealth should be treatment adherence rather than app adherence, as apps are no longer needed when goals are achieved.

### Purpose of the Study

This study is the last step in the information and communication technology (ICT) research conducted in MyCyFAPP. Clinical research applies the digital approach and evaluates its impact on quality of life [17]. Complementarily, the ICT perspective aims at developing an approach that best suits the needs of patients and HCPs and, from the evaluation of the approach, at deriving recommendations for CF care digital self-management, also filling the gap of knowledge about experiences from the digital self-management of chronic diseases.

Adopting a *design and creation* research strategy [18,19] and applying a user-centered design approach [20], our research followed an incremental process enabling to gradually understand problems and to improve solutions. Subsequent to the extraction of requirements [9], paper prototypes and mockups were cocreated with potential users [21]. Then, an initial software prototype was developed and tested in a midterm evaluation. Finally, an improved and comprehensive prototype was developed and evaluated in a 6-month clinical trial. This final evaluation addresses 2 research questions, the first related to user acceptance and the second to impact:

1. How do the software features and the context of use affect the user experience of the proposed digital self-management approach?
2. What are the perceived benefits and disadvantages of using this digital self-management approach?

We consider user experience broadly and investigate technical and nontechnical aspects that enhance or degrade the user experience. Understanding user acceptance and the impact of the approach is cornerstone for designing appropriate digital approaches. This represents the objective of this study.

## Methods

### Overall Approach

**Figure 1:**
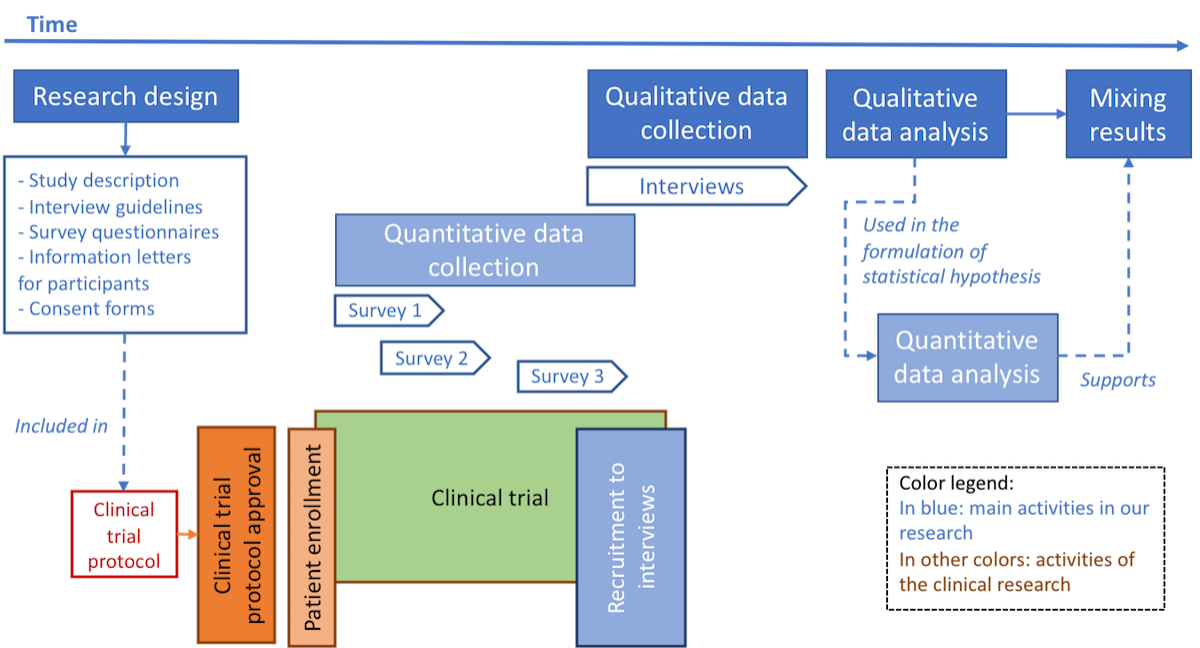
Flowchart of the research process.

Our study applied a mixed methods approach: an embedded design [22]. A flowchart depicting the design and implementation of the study is shown in Figure 1. As it had to be included in the clinical trial protocol, the study design was fixed [22]; methods were predetermined at the start of the research process, including constraints to avoid bias to the clinical study. The ICT research orientation was mainly qualitative, aiming at an in-depth understanding of system usage and experience. As usage over 6 months could not be observed, an interview approach was selected. Recruitment to interviews is often challenging and may affect results in unforeseen ways [23]. On the basis of earlier experiences with recruitment [9], convenience sampling was applied [24]. To reduce sampling limitations, 2 measures augment the findings: interviews of app users were complemented with interviews of HCPs about user feedback, and quantitative surveys were embedded in the self-management app to increase the credibility of qualitative findings [25]. Data collection was spread over time due to different start times in participating centers and spread patient enrollment. The collections of qualitative and quantitative data were independent. Qualitative analysis was performed before the quantitative analysis. The results were combined during the final step of the research process.

### MyCyFAPP Clinical Trial

A total of 171 patients from 6 CF centers in 5 countries (ie, Belgium, Italy, Netherlands, Portugal, and Spain) were recruited in the clinical trial, with most of them in a stable clinical status and with little or mild GI symptoms. A total of 154 patients completed the trial. Dropout causes were the addition of another task in the treatment or unexpected high enzyme dosage recommendations.

Patients and parents of young patients were asked to register symptoms and food intake, calculate the dose of enzyme, and consult the educational handbook. Each center appointed a dietitian for follow-up. Using PWT, dietitians monitored recorded data to check symptoms and diet quality and to compare enzyme intake with the dose recommended by the app. They sent messages to the patients’ app, including personalized advice and references to the educational handbook.

The clinical trial protocol established a minimum utilization of the app and the PWT. At months 1 and 6, food records had to be provided during 3 consecutive days, and symptoms were registered at least once a week. Otherwise, trial participants had to report at least three days per week and whenever needed (eg, new recipe). Using PWT, dietitians had to check patients and send feedback at least once a week. Depending on the dietitians’ criteria and patients’ needs, variations in the follow-up were allowed, such as the number of messages about educational content or the setting of nutritional goals.

### Qualitative Data Collection and Analysis

Due to the explorative nature of the research, individual semistructured interviews with open-ended questions were conducted, allowing interviewees to express their viewpoints freely [26]. We conducted 3 types of interviews (Multimedia Appendix 2):

1. With teens with CF and parents of children or teens with CF, covering motivation, usage, experienced impact, and future expectations
2. With HCPs about the self-management app, covering personal opinions and feedback from app users
3. With HCPs about PWT, covering usage and experienced impact on the workflow.

Due to the geographical distribution, interviews were conducted on the web (video call). Interviews lasted 45 to 60 min. Native language was spoken with Spanish and Portuguese trial participants; otherwise, English was spoken with the constraint that participants were fluent in English. Interviews were recorded, transcribed, and coded using the Dedoose tool provided by SocioCultural Research Consultants, LLC (Manhattan Beach, California). Data analysis was performed in an inductive manner following Klein and Myers’s framework [27]. The researchers performed a first independent round of thematic analysis, resulting in a number of initial topics [28]. Then, a refined set of topics was iteratively created through researchers’ collaboration.

Table 1 shows the target groups and informants across the CF centers. There were 41 interviews conducted involving 19 patients and parents and 12 HCPs; 10 HCPs participated in 2 interviews about the app and the PWT. The goal of including equal numbers per target group in all countries, thus equally covering countries that differ in the organization of CF health services and cultural backgrounds, could not be achieved.

**Table 1:** Overview of the target groups and informants across cystic fibrosis centers and countries.

Cystic fibrosis center	Self-management app: target group					Professional web tool: profession		Total informants
	Teens	Parents of young children	Parents of teens using the app	Dietician	Doctor	Dietician	Doctor	
Rotterdam	0	1	1	1	1	(1)^a^	(1)^a^	4 (6)^a^
Leuven	2	1+(1)^b^	(1)^b^	1	1	1+(1)^a^	0	7 (8)^a^
Valencia	2	1	1	1	1	(1)^a^	(1)^a^	6 (8)^a^
Madrid	2	1	1	1	1	(1)^a^	(1)^a^	6 (8)^a^
Milan	0	1	0	1	0	(1)^a^	0	2 (3)^a^
Lisbon	2	1	1	1	1	(1)^a^	(1)^a^	6 (8)^a^
Total	8	6+(1)^b^	5	6	5	1+(6)^a^	(4)^a^	31 (41)^a^

^a^Some health care professionals participated in 2 interviews, about the app and about the professional web tool. The second is registered in parenthesis.

^b^Parents of 2 children with cystic fibrosis, 1 young child and 1 teen using the app; only 1 interview was conducted.

### Quantitative Data Collection and Analysis

Questionnaires, developed in collaboration with clinical partners, were embedded in the self-management app. Standard methods, such as the system usability scale [29] or the technical acceptance model [30], were found to be too complex. Other concerns were a potential influence on participants and an additional burden. Thus, questionnaires were specifically developed for the study (Multimedia Appendix 2). They are kept short and inspired by standards. A psychologist from a CF center checked the wording.

Questionnaires were presented to users at 1 week, 1 month, and 5 months after starting using the app. The first questionnaire focused on usability and expectations and the others on user acceptance. To reduce intrusiveness and allow users to answer at any time, questionnaires were presented in a dedicated notification area rather than in pop-ups. Answering the questionnaires was not mandatory. The answers were anonymized.

Table 2 shows the response rates for each survey. As answers do not disclose the respondents’ center, we group centers by app language. Table 2 depicts high response rates for survey 1 (142/171, 83.0%) and survey 2 (120/171, 70.2% to 120/154, 77.9%) and decreasing response rates for survey 3 (84/154, 54.5%). Some factors identified through qualitative analysis may influence participation: knowledge acquisition made the app less relevant after a while, the trial duration undermined motivation, and the clinical status of participants was stable. In line with the possible ceiling effect of app usage, there were no significant differences in survey 2 answers between respondents to all questionnaires and respondents to only 2 questionnaires (all analyses ranged from t_118_=0.043 to t_118_=1.827 and with *p* values ranging from *P*=.06 to *P=*.98). Therefore, we do not use survey 3 in inferential statistics.

In the *Results* section, we present items on an ordinal Likert scale (% answered on given categories). However, inferential statistics assume the applicability of an interval scale.

**Table 2:** Response rates for the surveys.

Cystic fibrosis center	Clinical trial participants			Survey 1			Survey 2			Survey 3		
	Number of recruited participants	Number of dropouts	Number of participants who completed the survey, n (%)		Number of answers	Users, %		Number of answers	Estimated users^a^, %		Number of answers	Users, %
Rotterdam	17	2	15 (88.2)		53^b^	93.0^b^		46^b^	80.7-90.2^b^		37^b^	72.5^b^
Leuven	40	4	36 (90.0)		—^b^	—^b^		—^b^	—^b^		—^b^	—^b^
Valencia	25	0	25 (100.0)		46^c^	80.7^c^		36^c^	63.2-65.5^c^		20^c^	36.4^c^
Madrid	32	2	30 (93.8)		—^c^	—^c^		—^c^	—^c^		—^c^	—^c^
Milan	26	6	20 (76.9)		19	73.1		17	65.4-85.0		12	60.0
Lisbon	31	3	28 (90.3)		24	77.4		21	67.7-75.0		15	53.6
Total	171	17	154 (90)		142	83.0		120	70.2-77.9		84	54.5

^a^Some dropouts answered survey 2. The rate pertains to recruited users and users who completed the survey.

^b^Data from Rotterdam and Leuven are merged (language: Dutch).

^c^Data from Valencia and Madrid are merged (language: Spanish).

## Results

### User Experience With the Self-Management App

#### Outline for the Presentation of Results

This section presents the results of the qualitative analysis of interviews about the app and the quantitative analysis of surveys. Results from both analyses were found to be consistent. To avoid repetition, we aggregate the results and structure them according to the app features rather than to the research questions. First, we present descriptive statistics showing trends. Then, for each feature, based on the qualitative analysis, we address user acceptance (ie, how features were used and what could improve the user experience) and impact (ie, perceived benefits and disadvantages). Some inferential statistics for testing hypotheses derived from the qualitative analysis are described.

We use the terms *participants* for patients and parents involved in the clinical trial, *informants* for those involved in the interviews, and *respondents* for those answering the surveys. Illustrative quotes provided in Tables 3-7 are referred by *Id* in the text. As CF is a rare disease, we randomly number the CF centers to not expose the identity of informants.

**Table 3:** Illustrative quotes related to using the self-management app: varying motivation to use the app.

Id	Concern	Interviewee	Quote
M1	Motivation: health improvement	Mother of 6-year-old and 10-year-old children, C4	“Getting a little bit of weight. A lot less poo. You can see the nutrition. And you can see it helps. That is a big motivation. And you know that the enzymes are important. And you have to take the right dose. So that is a big motivation.”
M2	Motivation: self-efficacy	Parents of a 6-year-old child, C2	Father: “It gives us an answer. Those doses of Creon are rather useful. And surprising.” Mother: “It feels that we can now do something ourselves. It is not something we do because the hospital asked us. It is something we do because we want to help our daughter to improve her condition.”
M3	Lack of motivation: no patience, enough knowledge	Mother of an 11-year-old child, C3	“My daughter started looking at the app and said she had no patience for that and would not use it at school. [...] She does not want to bother with it as she already knows the enzyme dosage and do it herself alone, which is great.”
M4	Decreasing motivation: long trial	Dietician, C2	“In the beginning, most of them use the app a lot. Every month it became less, and less and less. For some, 6 months was really a long time.”
M5	Lack of motivation: limited time, break of routine	Dietician, C4	“We see that when they are going to school, they have like a structure and everything is going better, but when the summer starts, they are not that motivated anymore. Especially when they are going on holidays, when they are travelling, then they really don’t like using the app. Because it is time consuming and they don’t want to think of it.”

**Table 4:** Illustrative quotes related to using the self-management app: enzyme dose calculation.

Id	Concern	Interviewee	Quote
E1	Increased knowledge	Mother of a 5-year-old child, C4	“Before we just looked at the calories. Every 100 calories, we gave 1 “10 000” pills. But we learnt that, for the Creon, it is not as straightforward. For mayonnaise, we gave too little and now we know. After using the app, he gained much more weight.”
E2	Feeling confident	Mother of a 5-year-old child, C5	“At the beginning, I was really afraid because the doses were very high and I was scared to give this to my son, but then we decided to try. We were checking what we had entered to the app in the case it was very fatty. I felt comfortable because everything in the app is well measured. [...] Yes, I felt comfortable and there were no side effects.”
E3	Developing best practices	Mother of a 7-year-old teen, C1	“Sometimes I gave her X pills for a specific meal. But later on, she doesn’t want to finish up the dish. So, I wonder, what can I do? I learnt that I can give her some walnuts. It is a highly caloric food that she likes. Eating three walnuts is very easy for her, and it increases a lot the amount of Creon. I have learnt this thanks to the app. Or with the olives, it is the same.”
E4	Perceived benefits: gaining weight, better digestion	Dietician, C3	“Some increased weight. There was one patient that told us that finally he knows what normal faeces are. He said he had thought his faeces were normal, and finally they weren't.”
E5	Perceived benefit: child accountability	Mother of a 10-year-old teen, C4	“She is a teenager. Sometimes, she forgot to take her enzyme, but now she always takes it. We do not have to tell her...”
E6	Disadvantage: extra workload, but fewer symptoms	Parents of a 6-year-old child, C2	Father: “Yes, the app comes with extra workload. You have to do extra steps to calculate. But in the end, it makes it worthwhile to do it.” Mother: “Yes, we see when she goes to the toilets, it is much better. That’s worthy.”
E7	Behaviors in response to the recommended dose	Doctor, C2	“It was different. There were people who did not follow the advice and did just use their own usual dosage. And there were parents who did use the advice of the app. But, in general, if there was a big difference, they used to take their own dose.”
E8	Need for HCP^a^ support: wrong food recording	Dietician, C2	“Because sometimes they told “We got such a weird advice. I did everything right in the app, but we have to take 8 Creon for one piece of fish.” And then it turned out that they did something wrong, and if I filled it out again, it was only like 2 Creon.”

^a^HCP: health care professional.

**Table 5:** Illustrative quotes related to using the self-management app: nutrition management.

Id	Concern	Interviewee	Quote
N1	Adjusting diet	10-year-old teen, C4	“I looked at the calories often because then I could see how many calories I needed, and how many calories I was below the goal. [...] I drink more milk now for the breakfast.”
N2	Getting more disciplined	Mother of a 5-year-old child, C5	“The app helps you to be more disciplined with the food, and controlling nutrients makes you follow a more balanced diet. It has helped us to make his meals more balanced.”
N3	Nutritional goals as a game: educating young teens	Parents of an 11-year-old child, C6	“He eats less than necessary, and we put a lot of effort in pressing him to eat what is recommended by the dietician. So, the app helps our son to understand why we put a big pressure on him to eat. Sometimes it is very difficult to make him understand how important the amount of calories is. I would say it is like a game, you add your meals at the end of the day, you see the amount of calories and the distance to the goal. It was very useful in letting him understand he needs to eat more.”
N4	Need for HCP^a^ support: not reaching goals	Father of a 3-year-old child, C2	“We saw that it was quite difficult sometimes to get this right amount of energy, fat or things like that. [...] We checked with the nutritionist. She told us that it was OK with the things that we are giving him right now.”
N5	Disadvantages: obsession with goals, need for individual tailoring	Dietician, C6	“Yes, they liked it, but, in some cases, they were obsessed about the goals. Patients who have a good nutritionals status, like this information. They are concerned about the nutritional status. If the patients have problems with nutrition and see every day that they can’t reach the nutritional goals, it is bad. It is important to adjust the goals to every patient.”

^a^HCP: health care professional.

**Table 6:** Illustrative quotes related to using the self-management app: food recording.

Id	Concern	Interviewee	Quote
F1	Search: difficult in the beginning	Dietician, C4	“What I heard was that sometimes food products were difficult to find. The search function was not optimal. Especially in the beginning, it is quite hard and time consuming to know how to fill in everything and how to find the food. Once they were used to it and set their standards it was quite easy to register.”
F2	Technology literacy level	Mother of a 5-year-old child, C4	“It could be more user-friendly because I know if I would give that app to my parents... They are over 60. They would not know what to do... For us, it is OK because we find our way... But for older people, it would be too technical and too hard.”
F3	Food preparation, estimating quantities	Mother of a 15-year-old teen, C1	“The tedious part was the food recording. For example, there was an issue with the oil. When you created a dish and you indicated a certain amount of oil, then you could get a very high dose of enzymes, but if you changed the amount of oil the recommended dose could be reduced a lot. Also, it is difficult to estimate the amount of oil in fried products. Depending on the amount you indicated you could get too high doses.”
F4	Best practices: creating own dishes	Father of a 3-year-old child, C2	“You can create the meal in your app, so you select it the next time. We use that. Quite often. When the same meal comes back after 2 or 3 weeks, that was very pleasant to use.”

**Table 7:** Illustrative quotes related to using the self-management app: other features (health diary, educational handbook, and messages from health care professionals).

Id	Concern	Interviewee	Quote
O1	Health diary: more efficient consultation	Mother of an 11-year-old child, C3	“I actually found it useful. We try to register if our daughter goes to the bathroom or not, every day. Or if she has belly pain. [...] I think I used this most so that the doctor would know what happens with my daughter and so that I would not forget anything. Also the consultation was faster.”
O2	Health diary: a tool for reflection, increased well-being	Mother of an 11-year-old child, C3	“This part where you are asked if you feel well, happy, ... I ask my daughter in the evening if we can sit together and reflect about the day. It is just a click, and she finds it fun. It is the fastest part of all, the part she liked the most. Also, this makes us talk about school, the part I liked most [...] In these 5 minutes at the end of the day, we sat together and she told if she was happy, if she had had pain.”
O3	Educational handbook: better than the internet	15-year-old teen, C3	“And also the handbook. It helped because nowadays there is not much information which is specific in the Internet. Such as to explain the enzymes, how to improve things, which sports to practice. This you can’t find in the web easily.”
O4	Educational handbook: rather use the internet	Parents of a 6-year-old child, C2	“If we want to look up something, we will look for it on the Internet or call the hospital. There are so many other means to find information.”
O5	Educational handbook: useful to explain cystic fibrosis	Mother of 6-year-old and 10-year-old children, C4	“We read it. We like it a lot. [about 10yo child] She read it to and understood. She used the text from the app, but she made her own text and presented it to the class. And everybody understood.”
O6	Messages: personalized motivational messages	Mother of 6-year-old and 10-year-old children, C4	“The dietician sent us messages. “Very good. You do a good job.” My daughter was sick for a few weeks, and she [the dietician] sent some solutions: “Get a bit of pudding and milk and yogurt.” “You have to drink that and eat that. You are doing fine.””
O7	Messages: encouraging messages, but generic	Father of a 3-year-old child, C2	““Good job, thanks for filling in, you are doing good.” Yes, it was very positive. It was good to receive, but we thought it was some kind of computer. (laugh) To be honest. Sometimes it was the same messages.”
O8	Messages: lack support for messaging to HCPs^a^	Dietician, C1	“Some patients lacked the possibility to send messages to us through the app and PWT, which is something I would probably not include. It would be fine if the patients used it with moderation. Otherwise, something good can become something terrible.”

^a^HCP: health care professional.

#### Trends in Usability, Expectations, and Experiences

Table 8 depicts the usability results and expectations (survey 1) and the perceived user experience (surveys 2 and 3).

After 1 week, most respondents had positive experiences using the app. In total, 83.0% agreed or strongly agreed that the app is easy to learn and 70.4% agreed or strongly agreed that the app is easy to use. Respondents indicated very high expectations for the enzyme dose calculation (88.0%) and over half of them for nutrition management and understanding of the treatment. After 1 and 5 months, the perceived value slightly decreased compared with initial expectations and remained high for enzyme dose calculation (72.5% and 72.6%). The results are similar for months 1 and 5, except for motivation that further decreases. This decrease might be explained by similar factors as the decrease in participation rate (*Methods* section), that is, the rapid acquisition of knowledge, the trial duration, and the stable clinical status of participants. Throughout the trial, results related to enjoying using the app are stable (63.8%, 65.8%, and 63.1%).

The surveys included a multiple-selection question to identify features that were difficult to use (survey 1) and suggestions for enhancement (surveys 2 and 3). In survey 1, Respondents reported features related to food recording are most particularly difficult to use: MyCyFAPP dishes (reported by 21.9%), food diary (13.5%), and enzyme dose calculation (11.3%). Furthermore, around nine of ten of the suggestions (free text in the surveys) relate to food registration and almost half of those to additional food items. Suggestions are otherwise diverse and include support for medicine registration and app access from different phones allowing both parents to use their own phones.

**Table 8:** Percentage of respondents who agree or strongly agree with the claim and those who disagree or strongly disagree.

Claim	Week 1		Month 1		Month 5	
	Respondents who agree or strongly agree, %	Respondents who disagree or strongly disagree, %	Respondents who agree or strongly agree, %	Respondents who disagree or strongly disagree, %	Respondents who agree or strongly agree, %	Respondents who disagree or strongly disagree, %
I find the app easy to use.	70.4	11.3	Not asked	Not asked	Not asked	Not asked
I find learning to use the app easy.	83.0	5.0	Not asked	Not asked	Not asked	Not asked
I enjoy using the app.	63.8	10.6	65.8	6.7	63.1	8.3
The app will help (helps) me to find the right enzyme dose.	88.0	3.5	72.5	10.0	72.6	4.8
The app will help (helps) me to follow good eating habits.	Not asked	Not asked	54.2	9.2	53.6	10.7
The app will help (helps) me to understand the treatment.	66.2	7.7	55.0	10.0	53.6	9.5
The app will motivate (motivates) me to follow my treatment.	63.8	13.5	59.2	12.5	48.8	8.3

#### Variation Between App Users and Varying Motivations

Confirming our earlier research [9], a high degree of individuality in the manifestation of the disease and in patients is observed that affects needs, motivation, and perceived experience. The main influencing factors are health condition, knowledge about the disease and familiarity with the treatment, user behavior and personality (eg, compliance, structure, precision, curiosity, and reflection), and patient age.

Informants show various motivations for using the app, including expected health benefits, adjustment of enzyme dosage, tight follow-up by HCPs, and contribution to research (M1 and M2). Conversely, good health, experience and knowledge, personality, and age may reduce motivation (M3). Although HCPs expected patients with few GI symptoms to get less value from the app, most informants reported increased knowledge. On the basis of surveys 1 and 2, we tested whether positive anticipation after 1 week was connected to actual satisfaction after 1 month. Tests of correlation (Spearman R) show a moderately significant positive relationship between positive expectation and reported satisfaction (ρ=0.22-0.472; *P*<.001), meaning that respondents with positive expectations were more likely to report satisfaction. Although correlations are significant, they are moderate in size, meaning that a large amount of variation in satisfaction is related to factors other than positive anticipation.

HCPs described decreasing motivation along the trial and explained that increased knowledge and ability to self-manage and trial duration undermine motivation (M4). During vacation time, less structured than school time, patients were also less motivated (M5). Low motivation has a negative impact on the quality of records and, thus, their value for HCPs.

#### Enzyme Dose Calculation: The Most Appealing Feature

Enzyme dose calculation is the most innovative feature in MyCyFAPP as it supports the transition from learning about enzyme dosage through trial-and-error to an evidence-based method. Calculation support was indeed the feature most used and perceived as most useful. All informants from different target groups with more or less experience benefited from the feature. An analysis of variance test with Bonferroni post hoc tests showed significant differences between the reported satisfaction of the app features (*F*_3.476_=3.151; *P*=.03), with enzyme dosage being the highest (significantly higher than that for eating habits).

Beyond an accurate estimation of the dose, some informants learned about the dependency between enzyme and fat and some about the relationship between enzyme and symptoms (E1, E2, and E3). Reported benefits include improved health (in terms of gaining weight/height and reduced symptoms), increased confidence and self-efficacy, and child compliance to the treatment (E4, E5, and E6). The calculation is an additional task to the demanding treatment, but for most informants, benefits counterbalance this drawback. The informants acquired rapid knowledge about dosage and, due to stable diets, found calculation less useful after a few weeks. However, new types of food and diet changes during growth make calculation still relevant.

Despite an overall good experience, the calculation raised confusion when the recommended dose differed significantly from the usual dose. HCPs’ support was sometimes needed. Informants from Northern Europe reported changes in enzyme distribution throughout the day but similar total amounts for the whole day. In Spain and Portugal, some informants were recommended much higher doses than usual, and thus expected. Dosage differences between Northern Europe and Southern Europe were earlier found in a previous study conducted in MyCyFAPP [31]. HCPs recommended patients to adjust high doses to one-half or one-third, but informants handled recommendations differently, either giving it a try, adjusting to an intermediate dose, or ignoring it (E7). Although HCPs could set an individual correction factor for enzyme dose calculation, few made use of it due to lack of experience with the approach. Another related concern is the incorrect use of the app. Enzyme dose calculation requires food recording, and this was sometimes not done correctly (E8).

Interviews of HCPs in Italy indicate that the recommended doses were lower than usual, leading to a lack of motivation for using the app. In contrast to Spain and Portugal, no recommendation was given to adjust the dosage. On the basis of survey 2, we tested for differences between respondents in Italy and other countries (Table 9). Survey responses indicate that most respondents in Italy reported less benefit than others from using enzyme dose calculation (t_118_=2.216; *P*=.04) after 1 month. In addition, Italian respondents scored lower on motivation (t_118_=2.392; *P*=.02) and understanding of the treatment (t_118_=2.211; *P*=.03), but not on enjoying using the app or following good eating habits. The findings should be interpreted cautiously because of a low number of respondents in Italy.

**Table 9:** Comparison of responses after 1 month between Italy and other countries.

Item and country		n		Value, mean (SD)		*P* value		*t* test (*df*=118)	
**The app will help (helps) me to find the right enzyme dose**							.04		2.216^a^
	Others		103		4 (0.89				
	Italy		17		3.29 (1.26)				
**The app will help (helps) me to follow good eating habits**							.28		1.082
	Others		103		3.61 (0.92)				
	Italy		17		3.35 (0.86)				
**The app will motivate (motivates) me to follow my treatment**							.02		2.392
	Others		103		3.73 (0.96)				
	Italy		17		3.12 (1.05)				
**I enjoy using the app**							.38		−0.884^a^
	Others		103		3.78 (0.97)				
	Italy		17		3.94 (0.66)				
**The app will help (helps) me to understand the treatment**							.03		2.211
	Others		103		3.70 (0.89)				
	Italy		17		3.18 (0.95)				

^a^Indicates a significant Levene test for equality of variance.

#### Nutritional Management Toward a Balanced Diet

The follow-up of food intake was also perceived as a useful feature. Informants reported increased knowledge about nutrition and increased awareness about food intake, often leading to diet changes (N1 and N2). Few informants explicitly reported benefits from using the feature, except parents of small children who feel they were understanding and doing well, leading to less pressure on children (N3). However, interviews indicate that good nutrition has a high status. Several teenagers proudly reported that they adapted their diet. Acknowledgment of diet changes is sometimes needed from HCPs (N4).

On the downside, some participants found nutritional goals difficult to reach and experienced them as negative (N5). HCPs could set individual goals, but this was not done by all (preset goals depending on age were then used). In some centers, HCPs chose not to set goals for young patients, but parents felt that this decision was not well communicated.

#### Food Recording Needs to Be Simplified

Enzyme dose calculation and nutrition management require food intake recording. Many participants experienced recording difficult the first time they used the app, and, later on, they become a little efficient (F1 and F2). A combination of complex naming of food products and limited search functionality made the retrieval of products difficult. Beyond technical issues, other challenges included estimation of ingredients’ weights, specification of cooking method, and lack of knowledge about dish composition (F3). Most suggestions for app enhancements relate to food recording, for example, product barcode scanning and voice-based recording. Poor usability has led to incorrect recording and incorrect enzyme dose calculation. A dietitian explained that she was worried that some of the patients would not use the app properly, leading to incorrect enzyme intake. Indeed, it occurred in some cases (E8).

Functions facilitating recording were used by few respondents (F4). Training materials developed for HCPs to introduce the app use breakfast to illustrate the creation of *own dishes*. Most respondents created their breakfast, but no other dishes. In addition, some lacked usual dishes, despite the recommended country-specific dishes. These dishes were used by few, and informants reported that they failed to retrieve the dish composition in the app or lacked support for adapting dishes to personal habits.

#### Other Features Were Used to a Lesser Extent

Most informants only used the health diary at the start of the trial when asked to record bowel movement. Some reported that the diary was useful during the consultations for a more precise description of symptoms and that it helped them to reflect on their health status (O1 and O2). The need for a health diary decreased along the study as GI symptoms were reduced. Informants were willing to share data from health and food diaries, expecting these data will provide better insight to HCPs.

Opinions about the educational handbook vary. Some informants perceived the information to be better explained and more trustable than the information available on the internet, whereas others did not find it novel (O3 and O4). For many, the handbook was not a central feature. However, teens who need to learn more about CF or wish to explain the disease to peers may benefit from the information (O5).

The digital approach has an impact on the interaction between patients and HCPs. Informants appreciated receiving motivational messages, although some of them sometimes found messages a bit annoying (O6 and O7). Some lacked support for sending messages to HCPs, and, conversely, some HCPs had wished feedback on their messages (O8). Instead, contact with HCPs was taken by phone, email, or WhatsApp.

### Health Care Professionals’ Experience With the Professional Web Tool

The following results are based on interviews with HCPs. Dietitians appointed for follow-up during the trial worked intensely with the PWT. Most doctors used PWT less but still experienced it in actual settings and used it themselves. This section focuses on the reported benefits and disadvantages of PWT. Single features are not discussed specifically. In general, HCPs, most of them involved in the design, found PWT easy to use and useful. Preferences regarding features differed, but food records were highlighted as very useful by most of them, especially by dietitians. Illustrative quotes are provided in Tables 10-12.

**Table 10:** Illustrative quotes related to using the professional web tool: perceived benefits.

Id	Concern	Interviewee			Quote
B1	Patient information quality		Dietician, C4		“That the patient can record in the app and we can see like what they have eaten so that we have a very close follow-up of what the patient eats and also when he/she reports the Creon dosage we can see if it corresponds to the theoretical Creon dosage and also that we can see symptoms. If the patient report symptoms, like nausea or diarrhoea, we have close follow-up. We can see it and we can contact patients if we see abnormalities.”
B2	Identifying habits			Dietician, C1	“[Now, we] Really know what they eat. With the [paper food] records we used [before the app], it was harder to interpret. And, now it is more accessible. It is useful too, to see if they change eating habits depending on whether it's weekend, midweek, holidays; because this with the [paper] records is a bit difficult to see.”
B3	Patient information quality			Dietician, C6	“In some case that I have some doubts about what the patient tells me, I control and know the two information. It was helpful to control what they have told you yes, in some case it is important to have feedback from the PWT [...] No, I don’t think we have more information, maybe we have more correct information.”
B4	Personalized advice			Dietician, C1	*Do you think you are giving a better service?*^a^ Much better of course, especially personalized, which I think is what they most have noticed. Make it something for them, in real time and according to what they eat.
B5	Saving time: when contacting patients			Dietician, C2	“Sometimes it takes a lot of time because you call them in the morning, you get their voicemail, you leave a message in the voicemail, [...]. And in the afternoon, they have not called you back, so you call again. And every time before you call them you do check their dossier for how they are doing. So, you are actually reading on a patient again every time before you call them. And then they don’t pick up, I waisted another 5 minutes. It would be easy to communicate with the web tool for patients that really need that closer follow-up [...] And now I could see before the consultation already what they were eating, so it took me less time during the consultation to ask about that. Because I already knew a bit what their eating habits are.”
B6	Saving time: from paper records to digital records			Doctor, C3	*So during the trial you did not use the diet paper questionnaires you were used to?*^a^ “At the beginning yes, for comparing. But after, we did not use it, because we had the applications. It is much faster. Better data and faster.”
B7	Saving time: reduced workload before and during consultation, no longer need for manual calculation			Dietician, C1	*Do you think that the web tool helps you do your daily job?*^a^ “Yes, it makes it easier, more enjoyable, I save time. [...] The app does our function a bit. For example, it took half an hour to explain the dietary record from the last 3 months ago. Now the app tells it, but in real time. The content of the app is very useful because if the food record shows that the patient has not an adequate nutritional intake, he is redirected to the corresponding chapter in the educational content. And he does not search other sources, that is also important. Time is saved before consultation because the graphics show what is happening and it is easy to identify why. And then during the consultation, because you have already explained [the patient] with the app what he is doing wrong or good. [...] So above all, the nutritional control [is helpful], and for me the most important, the calculation of the nutritional intake, because up to now it was done using a manual spreadsheet. So I save time.”
B8	Saving time in yearly control		Doctor, C2		*Would it be useful for the yearly control?*^a^ “I think that would be very practical, yes. We now use food records on paper. If we had them electronically and could calculate automatically, that would be a good application.”
B9	Closer relationship, dialogue		Doctor, C2		“But if you really see the hard data, that is something you can really share with them, like “look what is happening”, you can start a conversation about it, it is not that you want to blame them but more “let’s see what is happening” and can we think together about the solution on how you can improve your compliance [...], but as a tool for a clinician it is great and it can really improve your practice and can give more information to have like a real useful conversation and to find more in partnership with parents and patients.”
B10	Tighter follow-up, closer relationship		Dietician, C2		*Do you know the patient better?*^b^*“*Not really, it’s just that you follow them more and are in more contact... Maybe that’s because they are participating in the study, that you are helping them with things, you reach out to them to ask about the app, how it is going. That’s the kind of stuff that makes the connection closer. I don’t really think that the connection is closer just because they use the app. Some of the patients I did not speak with a lot, the app was fine, they did not have questions.”

^a^Text in italics are questions to health care professionals.

**Table 11:** Illustrative quotes related to using the professional web tool: perceived disadvantages.

Id	Concern	Interviewee		Quote
D1	Useful with patients’ information		Doctor, C4	“[...] so, if they [patients] don’t fill it in, the clinician won’t look at it [...]; *Do you see an impact if the app was used in real practice?* ^a^ I think that it is only the case if patients often fill information. If not frequently used, then it is also difficult to say what the impact is on their daily life and whether it will reflect on compliance or whether you could use it for symptom control or looking for causes of abdominal pain.”
D2	Patients will not use app regularly		Doctor, C2	*What do you mean with “it has already a lot of impact in the daily life”?*^a^*“*Like the normal CF therapy, taking pills, doing nebulisation, doing physiotherapy, they have to do sports, think about Creon, so they have to think about collecting their medication in time at the pharmacy, if it is warm, they have to take salt supplements, ... Well it goes on and on and on. It is quite an organisation already. And, if you have more children or more children with CF, that is even harder to handle. If you then ask the parents “well, you just have to fill in the app once or twice a day”, it adds to all the other things that you need to do and look at yourself. You really have to think about how it impacts on the daily life of family, sometimes it is a real fulltime job. There are parents who stop working because of taking care of the children.”
D3	A close follow-up is time consuming		Dietician, C2	*Do you feel that the tool requires a lot of effort if you have to follow-up tightly?*^a^ “Yes. It does take a lot of time and now it is just 17 patients. But if all of our patients were using it which is like 150 patients. I could not send 150 patients a message twice a week.”
D4	A close follow-up is time consuming, extra effort for clinicians		Doctor, C2	*If you had these shared data, would you look at them between consultations or just during the consultation?*^a^*“*Maybe, if it was very easy to access, it would be quite useful, I think. *Also, between consultations?* [...] I think I would not look at the data when the patients are not coming for consultation. We don’t have time to do that; [...] it takes effort for clinicians to log in into the system and to look up all the data. [...] We have the luxury to have 20 min per patient, but still this is quite short, and we have to administrate everything and also talk to the parents, find a plan and execute your plan as well.”

^a^Text in italics are questions to health care professionals.

**Table 12:** Illustrative quotes related to using the professional web tool: interest in future use.

Id	Concern	Interviewee		Quote
I1	Future frequency of use—daily		Dietician, C4	*Would you like to use in the future?*^a^ “Yes, off course, I really like it [...] I would like to use the PWT on a daily basis.”
I2	Future frequency of use—monthly		Dietician, C2	“But I would not use it twice a week to send messages. *You would not follow either if they are registering things?* ^a^ Not as much as I did during the trial now. Maybe once a month or before they come to the hospital.”
I3	Gaining weight, newly diagnosed		Dietician II, C4	“I would check it like on a weekly basis and also when I know when some patients have trouble gaining weight that I will follow them closer, or when there are new diagnosis that I also can follow them closer like when they are home, I would use it more for the kids who have problems or if parents ask me to check.”

^a^Text in italics are questions to health care professionals.

#### Positive Impact on Health Care Services and Workload

The main benefit HCPs reported is that PWT allows them to obtain more information about patients, for example, regular information about symptoms, eating habits, and enzyme intake. This information facilitates a closer follow-up because the more information HCPs have access to, the easier it is to compare it with other insights about patients and to interpret it. Correlations between eating habits, medicine intake, and symptoms can be detected, and HCPs can react quickly when noticing adverse symptom development (B1). Furthermore, HCPs highlighted that continuous monitoring provides a better overview of patients’ behaviors. Within regular visits every 3 months, HCPs only get a small glimpse or a very general overview of patients’ eating habits and health status. PWT allows a fuller picture of the course of the disease and the patients’ management of it (B2).

HCPs also reported that information communicated by patients is more reliable and accurate. Normally, patients do not record data systematically. They forget details or get information mixed up. Empowering patients to record events at the time they occur, HCPs felt that the data they receive are more precise and better reflect the reality of patients’ status (B3). Having more and more precise information during the trial, some HCPs reported that they were able to give better and more personalized advice (B4).

Not only do HCPs think that they can deliver a better service using PWT but they also see a positive effect on their workload. Using PWT, the time needed for consultations with patients can be reduced. One HCP reported that she was able to get in contact with patients more efficiently (B5); others reported that information can be analyzed quicker than when using paper food records (B6). In particular, dietitians looked at the registered information beforehand and spent less time on asking for symptoms or eating behaviors (B7). PWT can also help to make work easier. For instance, dietitians collect and assess food records on a regular basis. This process becomes easier with the digital approach (B8).

Furthermore, some HCPs experienced that PWT facilitated new positive interactions with patients. On the basis of the registered information, an HCP reported that it was possible to start a more objective dialogue with patients and to involve patients more intensely in their treatment (B9). Several HCPs reported that relationships with patients became closer. However, they were unsure if this was only due to the frequent interactions required by the clinical trial protocol or due to the messaging support in PWT (B10). Nonetheless, HCPs perceived this closer relationship to be positive. Not all HCPs noticed a change in the interaction with patients. Some HCPs reported very good relationships with patients, with or without the approach.

#### Drawbacks Related to Available Information and Workload

The HCPs also reported about challenges and disadvantages they experienced using PWT. One notable challenge is that the usefulness of tools depends strongly on the data entered by patients. If patients do not record data or record incomplete or incorrect data, PWT is of little or no use (D1). HCPs were worried that it can be a burden for patients and parents to record data and did not expect all patients to fill in information, at least over a longer period (D2).

Some HCPs also reported an additional effort with a close follow-up using PWT. In the clinical trial, with resources specifically allocated for this task, HCPs were able to invest extra effort, but they did not expect to be able to continue such a close follow-up in their regular practice for a higher number of patients (D3 and D4). This may seem contradictory to the earlier described reduced workload. If we take into account that HCPs were asked to communicate with patients much more often than they were used to during the trial, PWT itself does not necessarily mean more work for HCPs, but the tool offers new means of interaction that, depending on the self-management approach, can imply more effort for HCPs than current procedures.

#### Potential Future Use of Professional Web Tool

Most HCPs were interested in using PWT in the future, though they have very different ideas regarding the frequency of use. Some think of using the tool daily, and others are only interested in using the tool on a weekly or monthly basis (I1 and I2). As a close follow-up of all patients would be time consuming, some HCPs suggested using PWT for specific patient groups or using specific functionalities. This would reduce their own and their patients’ efforts. Specific patient groups would be those who generally need a closer follow-up or those who need tight support for a limited period, for example, newly diagnosed children and their parents and patients currently not feeling well (I3).

## Discussion

### Principal Findings

Our results show a positive experience of the proposed self-management approach. They indicate that challenges relate more to human factors, context of use, and lack of experience with digital self-management than to technology. Confirming previous findings, technology literacy level [32-34], positive reinforcement [35], and contextual factors [36] influence the user experience. Patients get less eager to use the app when they have reached their goals [16,36].

Adopting a user-centered design approach, we expected the app and PWT to be easy to use and useful. They mostly are, with the exception of food recording. Feature acceptance varies, reflecting the diversity of needs that we and others previously discussed [9,15]. Enzyme dose calculation was the most useful. In addition, users benefited from nutrition management. Patients and parents increased their awareness about nutrition, and HCPs collected more reliable patient data. Patients and parents were able to rapidly increase self-efficacy. They adapted enzyme intake and nutritional habits when needed, and some reported enhanced quality of life. For HCPs, rich and reliable data enabled personalized follow-up. Although tight patient follow-up may require additional workload, messaging and digital food recording save time.

Similar to other studies, our study shows the importance of trust in information (eg, educational handbook) [37,38]. In contrast to other studies, privacy was not a concern in our study [39,40]. Patients who already used to tell HCPs about their behaviors were willing to share data and expected them to be useful for follow-up.

### Recommendations

On the basis of these findings, we propose recommendations for the successful adoption of digital self-management. As there is an overlap between our findings and those from previous research, it is relevant to also consider these recommendations for self-management of other chronic diseases in addition to CF.

#### Identifying Relevant Use Cases

Motivating patients to use health apps over a long period is a recurrent topic in mHealth. The purpose of self-management is, however, to support the acquisition of self-efficacy skills [41], not to motivate for using an app. Despite the relatively good clinical status of trial participants, many benefited rapidly from using the app. Over time, as participants acquired new skills, motivation tended to decrease, leading to poorer registered data. Rather than seeking motivational approaches to keep using the app, we propose to identify relevant *use cases*, that is, patient groups and situations where patients are most likely to benefit from using the app. From a professional viewpoint, use cases would reduce the workload.

Obviously, the worsening of symptoms and the occurrence of new symptoms are a relevant use case, as symptom descriptions and food intake provide HCPs a basis for diagnosis and recommendations. HCPs suggested focusing on learning cases, such as parents of young patients and adolescents. The latter case gave positive results for juvenile arthritis [42]. As we observed that patients with few symptoms can benefit from using the app, using the app a few days before the regular consultations or in connection with the annual check [8] is also a relevant use case.

#### Personalizing and Supervising

The need for personalization is often discussed in connection to technology [43-46]. Our study also identifies the need to personalize the introduction of technology. Technology literacy level and capacity to learn, seek new insights, and reflect have an impact on the adoption of solutions. Supervision by HCPs is, therefore, essential when introducing digital self-management and should be tailored to patients’ skills. Our study also indicates the importance of a regular follow-up by HCPs for motivation.

Advice should be provided to patients during consultations or between consultations using messaging. For example, advice may relate to dealing with unusual enzyme doses to explain details that are easy to overlook for correct calculation, for example, fat registration and food amount. Some patients need support to use the app efficiently and effectively, for example, creating dishes. Some need guidance for exploring features and understanding their rationale, for example, relating symptoms to food and enzyme intake or retrieving hints about nutritional goals in the educational handbook.

Patients should be gradually introduced to the app features. In our study, all features were presented to patients and parents at the start of the trial. The app is comprehensive, and we observed that some users were not aware of all features. A gradual introduction can be addressed technically. The ultimate goal is to reduce the need for follow-up by HCPs to support patients in countries where health care services are limited. The features could be activated gradually, and patients provided hints. For example, the app could detect an unexpectedly high number of calories in food recording and notify the user.

#### Developing Health Care Professionals’ Skills

As digital self-management is a new practice, HCPs need to develop new skills. Although HCPs were trained to teach patients to interact with the app, our results indicate that tutorials should cover guidelines about the process, for example, gradual introduction to features and advice for app exploration. Best practices developed by parents should be included in tutorials, for example, dealing with a child who eats less than expected or speeding up food recording.

Beyond technical aspects, HCPs need advice for collaborating with patients. Most importantly, HCPs should agree with patients about the frequency and type of follow-up. They should also clarify what parts of self-management are relevant. Similar to the prescription of a medication, app usage should be adapted to patients. For example, nutritional goals may be stressful for some individuals and may be irrelevant for young patients. Dietitians were found to be more or less skilled in writing motivational messages. Practices can be collected and shared between HCPs. The availability of predefined messages in PWT should be reconsidered. Automatic generic health messages may appear as depersonalized and weakened relationships with HCPs [47].

#### User-Centered Design in the Development of Content

Trial participants reported the complexity of product names in the app. Although software development adopted a user-centered design approach, food databases were developed by dietitians without involving users. In addition, food databases in native languages were not ready for testing at midterm evaluation. As digital solutions are increasingly being developed in clinical settings, user-centered design is a software engineering practice that HCPs should become familiar with.

### Future Research

Digital food recording is generally complex, with a trade-off between usability and accuracy. Technology for food image recognition was not yet accurate enough when we developed the app [48]. Recent solutions still seem too inaccurate for the purpose of enzyme calculation.

The approach to enzyme dose calculation necessitates refinement for optimal support. Guidelines for setting the individual correction factor as well as for specifying the cooking method are needed. As the enzyme dose mainly depends on the amount and type of fat, simplification of food recording should be investigated. Simplification may, however, act to the detriment of an increased knowledge about nutrition.

### Strengths and Limitations

For practical and economic reasons, interviews were conducted on the web. Video calls were set up to reduce the *distance* between the interviewee and interviewer. In some countries, English was used rather than the native language. Using English was preferred over involving an interpreter because of the explorative approach of interviews. No language barrier was experienced during the interviews.

The clinical trial context does not truly represent the regular clinical context. The tight follow-up by HCPs and the commitment of trial participants to contribute to research may have influenced the app and PWT usage. Still, the research allowed us to identify usability shortcomings and factors relevant in clinical contexts. Deployment in a real context is needed to strengthen the insights about organizational issues.

Although the clinical protocol specified requirements on app usage, it left trial participants some freedom, leading to differences in the way the app was used. HCPs also had different expectations for the approach and were more or less acquainted with mobile technology. HCPs used PWT more or less often, and messaging was performed in different ways. This influences the support provided to patients. Such differences would, however, also apply in a regular clinical context. Most importantly, extracting practices that lead to positive outcomes and building guidelines upon them are needed.

## Appendix

Multimedia Appendix 1 Features and screenshots.

Multimedia Appendix 2 Questionnaires and interview guidelines.

## Abbreviations

CF: cystic fibrosis
GI: gastrointestinal
HCP: health care professional
ICT: information and communication technology
mHealth: mobile health
PWT: professional web tool
