# Supplementary material for: Users’ Experiences of a Mobile Health Self-Management Approach for the Treatment of Cystic Fibrosis: Mixed Methods Study
Source: JMIR Mhealth Uhealth. 2020 Jul 8;8(7):e15896. doi: 10.2196/15896 (PMC7381063; doi:10.2196/15896)

Multimedia Appendix 1: Features and screenshots

MyCyFAPP self-management app

The self-management app targets parents of young children with CF and teenagers with CF. The following features are illustrated through screenshots: summary of today's recorded information; health diary for recording mood and GI symptoms; food diary for food recording and follow-up of food intake; calculation of a personalized enzyme dose depending on meal composition; educational handbook about the disease and the treatment with focus on nutrition.

In addition, other features are supported: access to MyCyFAPP dishes, a compilation of country-specific dishes, created specifically for the app, aiming at correcting specific nutritional imbalances; graphs for visualising the nutritional and health status over time; reminders for medication intake, medical appointments, or any other relevant due task; sharing of recorded data with the Professional Web Tool (PWT); retrieval of information set by HCPs on the PWT: nutritional goals, individual correction factor for the enzyme dose calculation and messages.

Today's summary

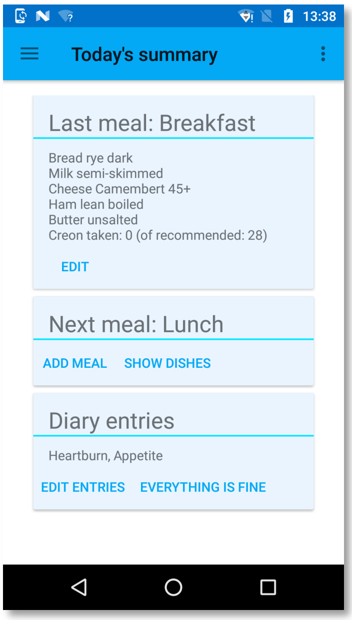

Health diary: overview and recording example for bowel movement

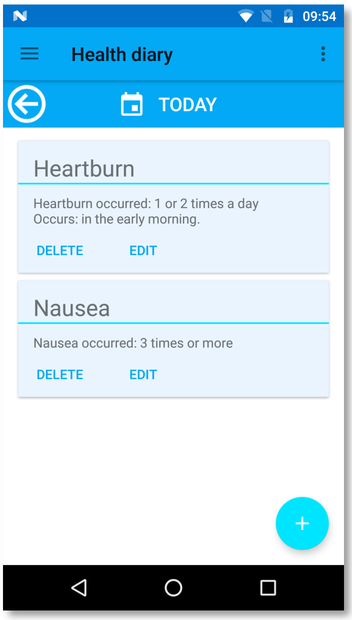

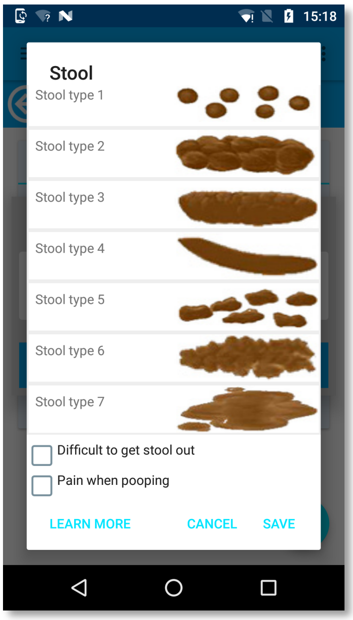

Food diary: overview, recording a dish and food group classification

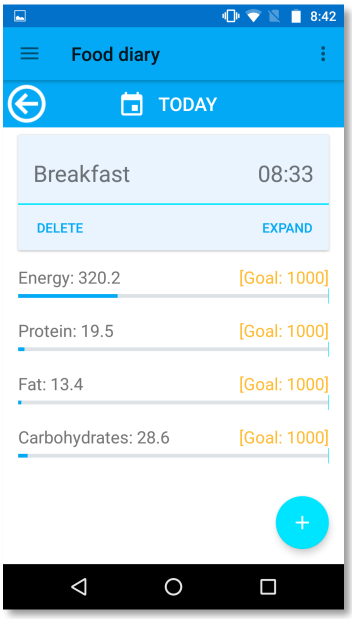

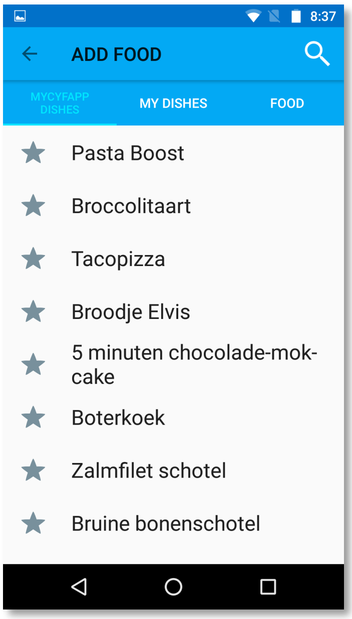

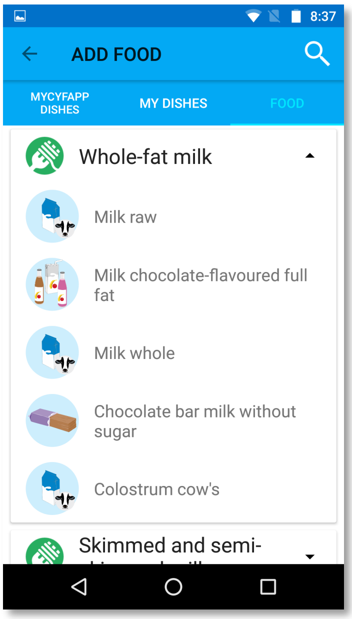

Enzyme dose recommendation and educational handbook

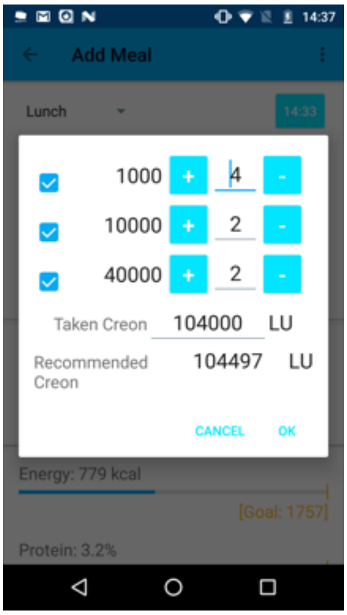

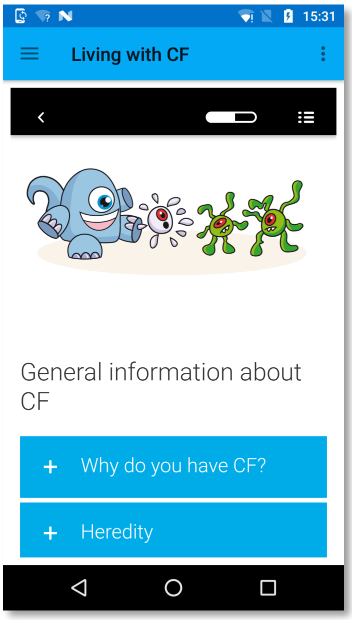

MyCyFAPP Professional Web Tool

The Professional Web Tool (PWT) targets HCPs. It provides an overview of the progress of the patients, mainly based on the data recorded using the self-management app. Some information is also gathered during consultations, e.g., weight and height. The monitored parameters are those included in CF nutritional guidelines: nutrient intake, enzyme dose and nutritional status. The tool entry point is the patients' list, from where HCPs can get access to patient information that consists of a Patient Profile and Patient Records. Through the Patient Profile, HCPs can register health information, set nutritional goals and send messages to patients. Patient Records provide a visualisation of the data recorded by the patients.

Patients' list screen

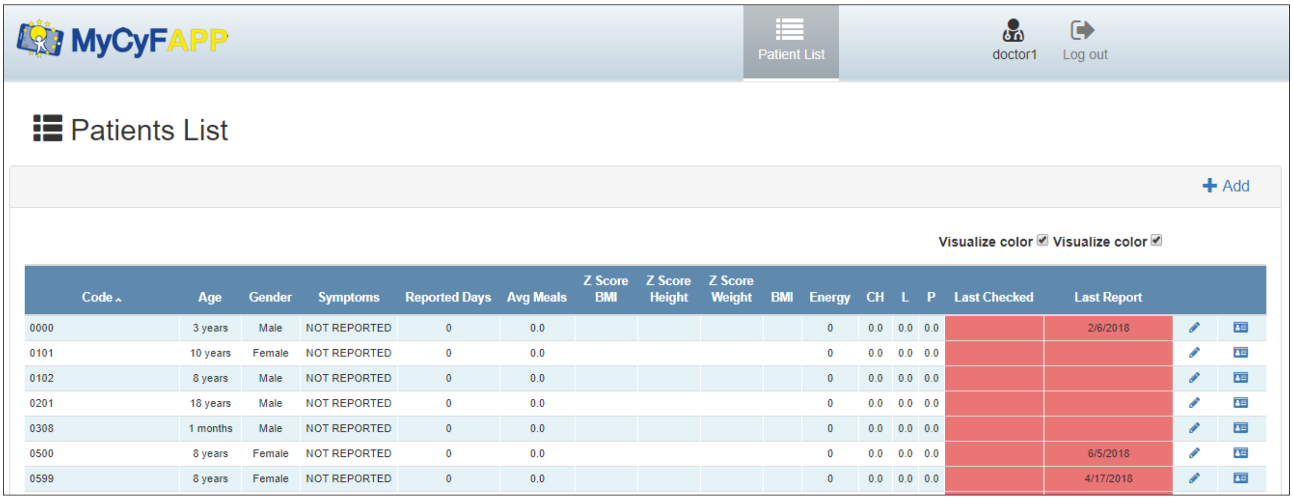

Patient profile (upper part) and health profile with GI characteristics (lower part)

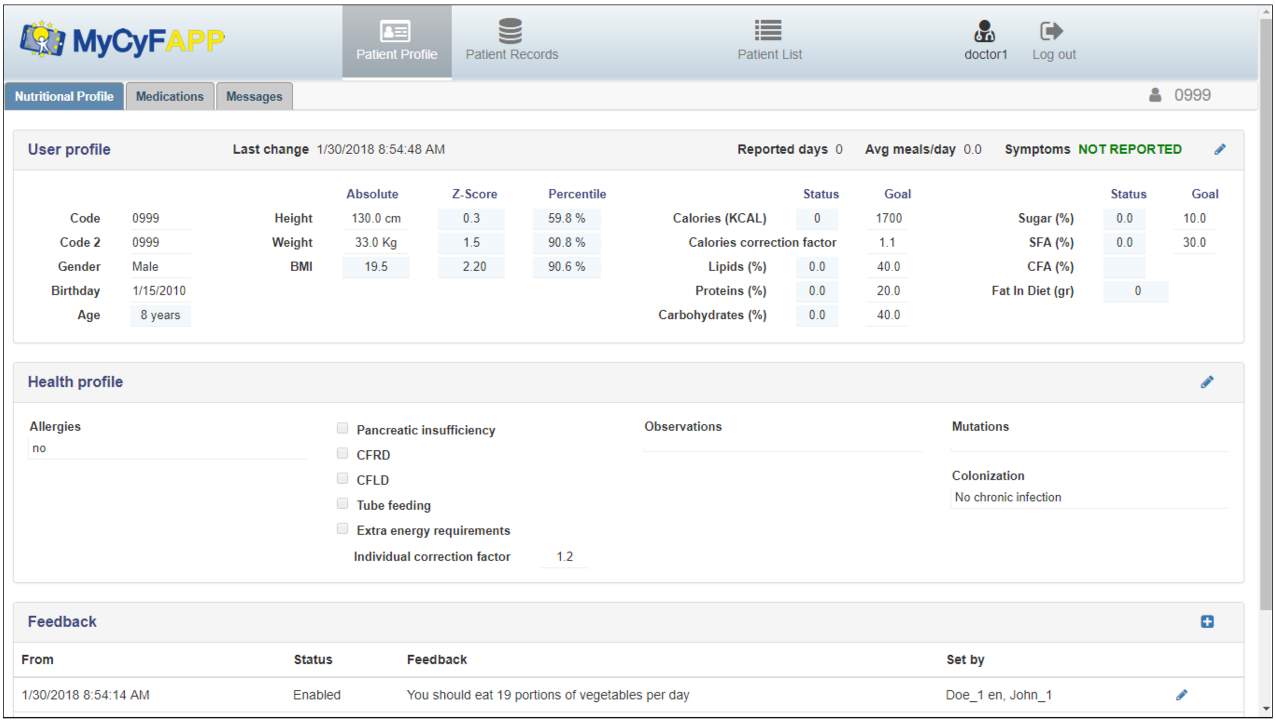

Patient profile (upper part) and medication list (lower part)

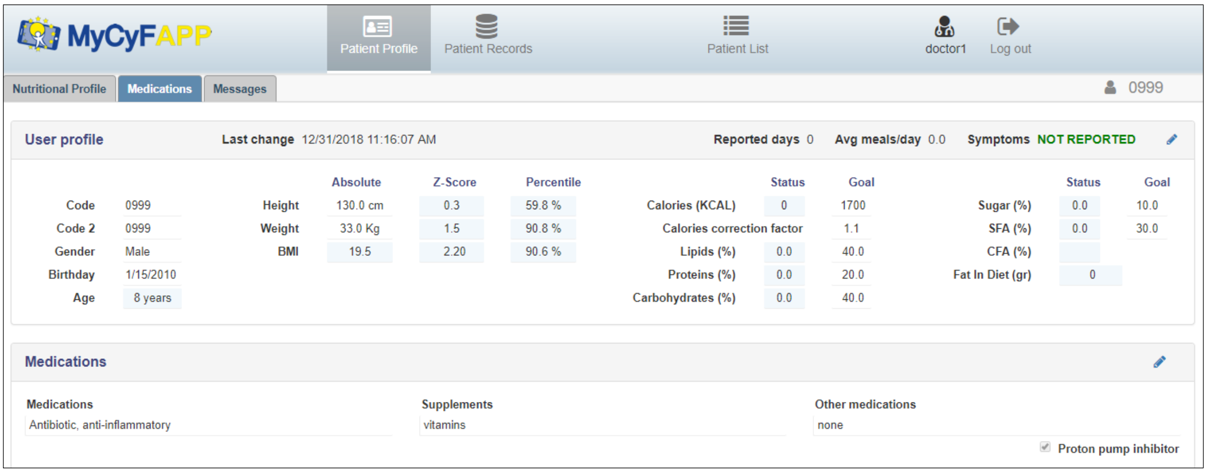

Patient profile (upper part) and recommendation messages (lower part)

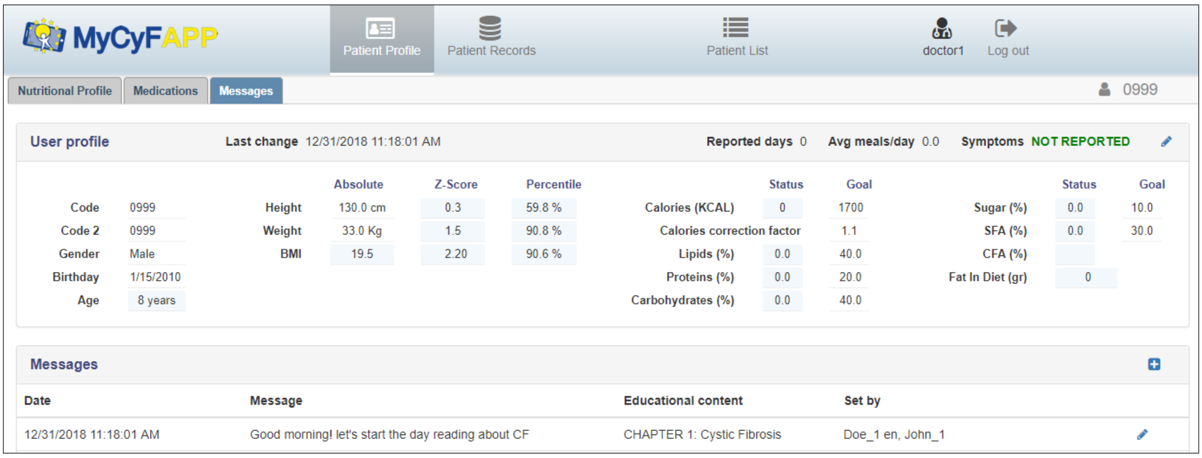

Patient records: nutritional intake

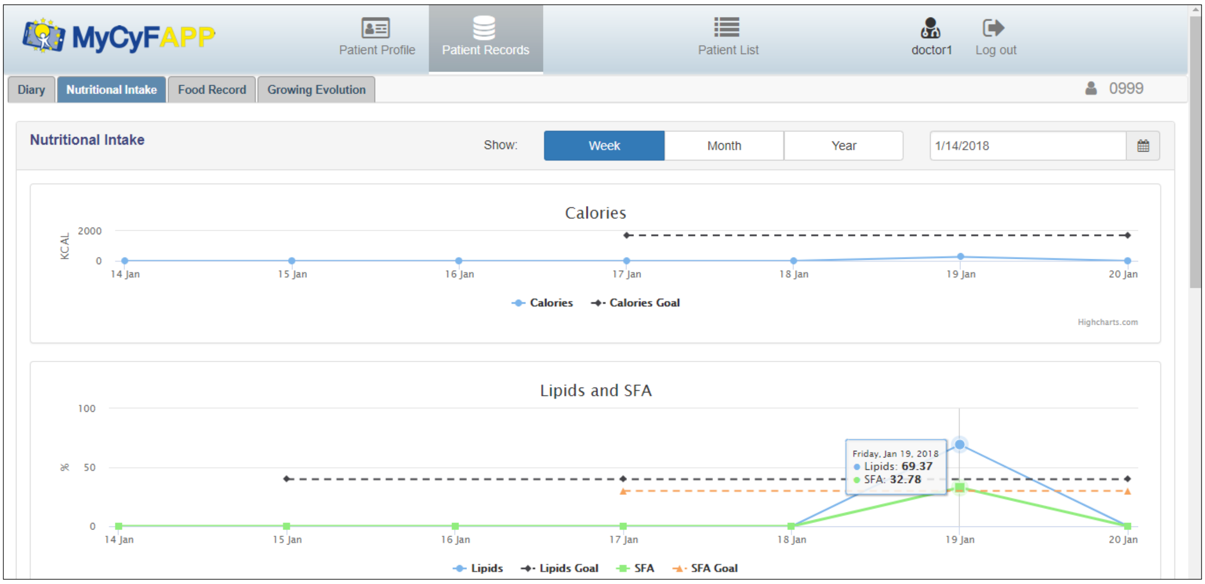

Patient records: food records over time

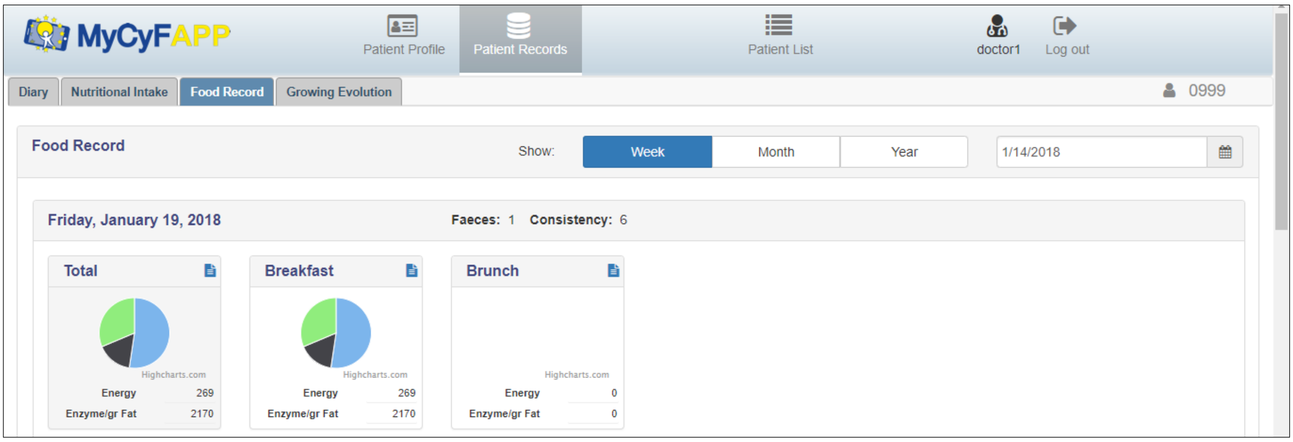

Patient records: growth in percentile or Z-score scales

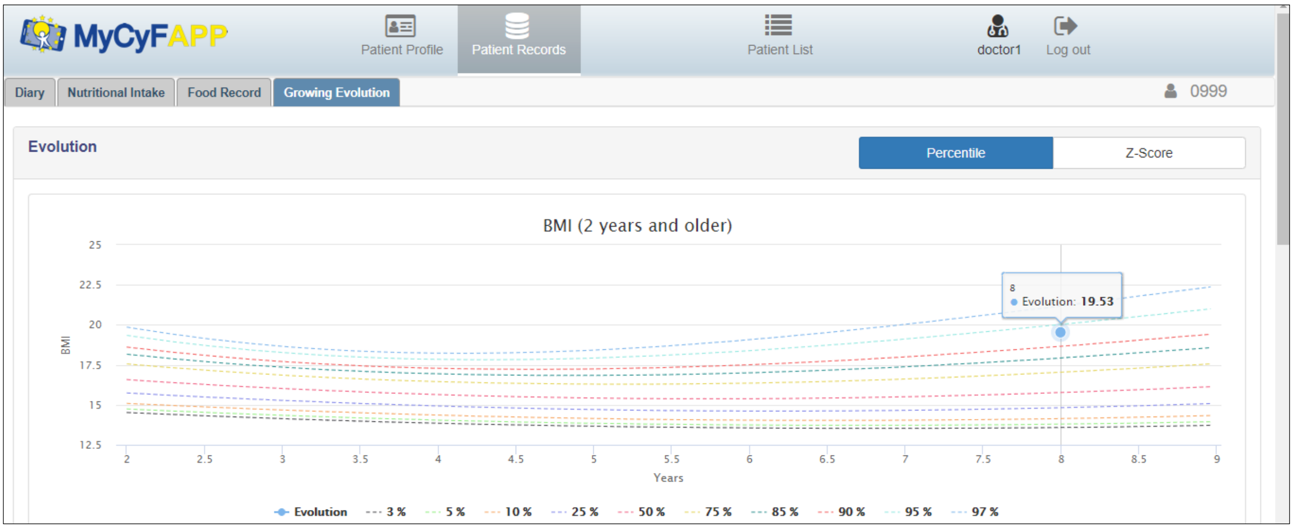

Patient records: health diary including mood and GI symptoms

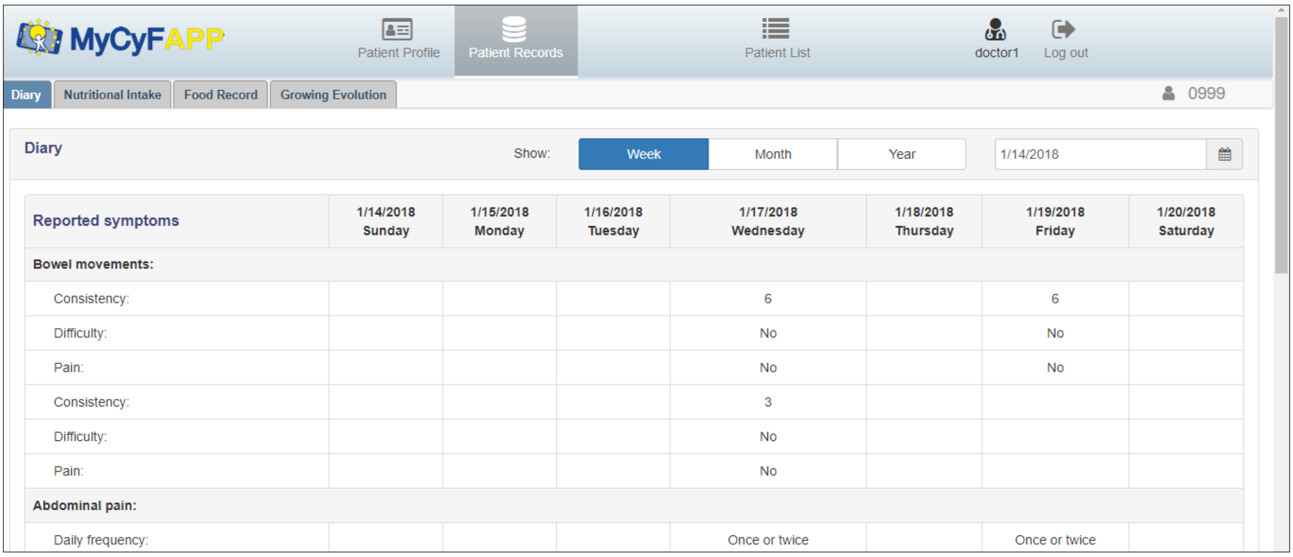

Supplement: Multimedia Appendix 1 [file mhealth_v8i7e15896_app1.pdf]
