# Supplementary material for: Users’ Experiences of a Mobile Health Self-Management Approach for the Treatment of Cystic Fibrosis: Mixed Methods Study
Source: JMIR Mhealth Uhealth. 2020 Jul 8;8(7):e15896. doi: 10.2196/15896 (PMC7381063; doi:10.2196/15896)
Supplement: Multimedia Appendix 2 [file mhealth_v8i7e15896_app2.pdf]

Multimedia Appendix 2: Questionnaires and interview guidelines

Questionnaires in the self-management app

Questionnaire 1 (after 1 week): Focus on usability and expectations

Question	Response type
I find the APP easy to use.	Five-level likert scale
I find learning to use the APP easy.	Five-level likert scale
I enjoy using the app.	Five-level likert scale
I find the following features difficult to use:<br>Today's summary; Food diary, Enzyme dose calculation; Dishes; Goals; Health diary; Living with CF; Reminders; Charts	Select from list<br>(0 or more)
I expect that the APP will help me to find the right enzyme dose.	Five-level likert scale
I expect that the APP will make it easier to understand the treatment.	Five-level likert scale
I expect that the APP will motivate me to follow the treatment.	Five-level likert scale

Questionnaire 2 (after 1 and 5 months): Focus on acceptance

Question	Response type
I enjoy using the app.	Five-level likert scale
I find the following features particularly useful:<br>Today's summary; Food diary, Enzyme dose calculation; Dishes; Goals; Health diary; Living with CF; Reminders; Charts	Select from list<br>(0 or more)
I miss the following features:	Free text
The APP helps me to find the right enzyme dose.	Five-level likert scale
The APP helps me to follow good eating habits.	Five-level likert scale
The APP helps me to understand the treatment.	Five-level likert scale
The APP motivates me to follow my treatment.	Five-level likert scale

Interview guidelines

The following guidelines were developed:

Interview guidelines for parents and patients (self-management app)

Guidelines for Health Care Professionals (self-management app)

Guidelines for Health Care Professionals (Professional Web Tool)

Interview guidelines for parents and patients (self-management app)

Context

- About you: your age / age of your child
- (for parents of chidren < 12): Was your child using the game?

About usage

How and when did you use the app?

- What features did you use? Why?
- How did you like the app?
- How did you like the different features? *Recall list of features*
- Did you get messages from the doctor/dietician through the app?
    - If so, what was the purpose with the messages (e.g., encouragement, advice, reminder)?
- What features should be improved? Why?
- What features did you miss?

(Only for teens) Did you use the app yourself or with your parents?

Did you use the app regularly?

In which situation? When? Where?

- When did you use the app? You can help answering giving examples:
    - At the end of the day?
    - When things happen? (e.g., at mealtime, when having a symptom, during consultation)
    - When the doctor asks you to do so?
- Where did you use the app? (e.g., at home, at school, in the bus)

Did the dieticians or doctors help you to use the app?

About impact/experience

- Overall, what was your motivation for using the app?
- Any particular situation that motivates you to use the app? (e.g., new type of food, stomach ache, question during consultation)
- Did you experience any positive or negative effect of using the app?
- Did you feel confident that you were following the treatment well? Why?
- Did the app change the way, the treatment was followed?
- What about estimating the enzyme dose?
    - Did you find it difficult before using the app? Did the app help you?
    - Do you still need to calculate after using the app for 6 months? Or do you manage it by yourself?
- What about eating habits?
- Were you worrying about food before using the app? Did the app help you to acquire good eating habits?
- Did you improve your understanding of the treatment? How?
  (For parents) Did it improve the way you explain the treatment to your child?
    - What about education materials?
    - What about self-reporting?
- How do you feel about sharing data recorded in the app with doctors?
- Did you change the way you talk to the doctor or dietician during consultations?
    - Do you use the app to remember what happened? E.g., what symptom you had.
- Did you change the way you communicate to the doctor or dietician between consultations?
- Any other effects of using the app?

About expectations

- Would you like to use such an APP in the future?

Interview guidelines for Health Care Professionals (self-management app)

Context

- Country, name of CF Center
- Profession and position in the CF team:
- How many patients were included in the clinical trial at your center?
- How many drop outs were there? When? Why?

General feedback of patients and of parents on the self-management app

<u>Please try to leave your personal opinion out for the first part of the interview.</u>

- What feedback did you get regarding the self-management app?
- Overall, do you think they liked the self-management app or didn't like the self-management app? Why?
- What did they like about the self-management app?
- What didn't they like about the self-management app?
- What problems/issues regarding the self-management app did they report during the trial?
    - Technical? Contextual? (e.g., at school, at home…) Others?
- What features did they like most? Why?
- What features did they like the least? Why?
- What did they think of the rest of the features? *Recall list of features*

User behaviour

- Did you get the impression the self-management app was used a lot or only seldomly?
- How often do you think the self-management was used on average? Daily? Weekly? Monthly?
- Did the usage of the app change over time?
    - Were the users more interested in the self-management app in the beginning then later on in the trial? Or the other way around?
    - When did you notice the change?

Perceived Impact / Usefulness

- What benefits of the self-management app were reported?
- What downsides of the self-management app were reported?

Tips/ Outlook

- Did patients and parents make suggestions for changes? What were they?
- Did patients and parents make suggestions on what should stay the same?

Perspective of the Health Professional

- What was your experience with the self-management app during the clinical trial?
    - What was good about the self-management app? What did you like?
    - What wasn't good about the self-management app? What didn't you like?
- What features did you like? What features didn't you like? *Recall list of features*
- Was it necessary for you to motivate patients to use the self-management?
    - If so, why do you think this external motivation was necessary?
    - If so, what motivation approach did you use?
- Did you notice an impact of the self-management app for patients and/or parents?
    - Do you think there was a learning process/knowledge increase with your patients?
    - Did the self-management app help with the interaction with parents and patients during consultations?
    - Did the self-management app help with the communication with parents and patients between consultations?
    - Did you see improvements regarding the treatment?
    - Others?
- Did you notice an impact of the self-management app for your work? Was it helpful for your work?
    - What impact did you see?
    - Did you use the app with patients and parents during consultations? Was it of help?
- What would you change about the self-management app?
- What should stay the same?

Guidelines for Health Care Professionals (Professional Web Tool)

Context

- Country, name of CF Center
- Profession and position in the CF team:
- How many patients were included in the clinical trial at your center?
- How many drop outs were there? When? Why?

General feedback on the PWT

- What do you think of the PWT after using it during the clinical trial?
- Overall, did you like using the PWT?
- What did you like about the PWT?
- What didn't you like about the PWT?
- Was there any inconsistency in the system?
- Was it well linked with the self-management app?
- Did you find PWT easy or cumbersome to use? Did you have any kind of problems/issues regarding the PWT during the clinical trial?
- What features did you like the most?
- What features did you like the least?
- What do you think of the rest of the features?

Behaviour

- How often did you use PWT normally? Almost never? Once per week? Twice per week?
- Did your usage of the PWT change over time?
    - Did you use it more often in the beginning then later on in the trial? Or the other way around?
    - When did your usage change?
- When did you use PWT?
    - Before the consultation? During the consultation? After the consultation?

Perceived impact/ Usefulness

- What benefits of the PWT did you noticed?
- What downsides of the PWT did you notice?
- Did you notice an impact of PWT for your interaction with patients and/or parents?

- Did PWT help with the interaction with parents and patients during consultations?
    - Did PWT help with the communication with parents and patients between consultations?
    - Did you see improvements regarding the treatment? Was it more personalized?
    - Did you think PWT has helped to provide better service to parents and patients?
    - Do you know better knowledge about the patients?
    - Others?

- Did you notice a specific impact of the PWT for patients and/ or parents?
- Did you notice a specific impact of the PWT for your work?
    - Was it helpful for your work?
    - What impact did you see?
    - Did you feel confident of using it?
    - Did you integrate the tool in consultations? Was it of help?
- Any other effect you noticed while using PWT?

Tips

- Do you have suggestions for changes? What are they?
- Do you have suggestions on what should stay the same?
- Would you like to use PWT in the future?
